# Factors Affecting the Reproduction and Mass-Rearing of *Sclerodermus brevicornis* (Hymenoptera: Bethylidae), a Natural Enemy of Exotic Flat-Faced Longhorn Beetles (Coleoptera: Cerambycidae: Lamiinae)

**DOI:** 10.3390/insects11100657

**Published:** 2020-09-24

**Authors:** Costanza Jucker, Ian C.W. Hardy, Serena Malabusini, Silvia de Milato, Giacomo Zen, Sara Savoldelli, Daniela Lupi

**Affiliations:** 1 Department of Food, Environmental and Nutritional Sciences (DeFENS), University of Milan, via Celoria 2, 20133 Milano, Italy; costanza.jucker@unimi.it (C.J.); Silvia.demilato@unimi.it (S.M.); Giacomo.zen@studenti.unimi.it (S.d.M.); Serena.malabusini@unimi.it (G.Z.); sara.savoldelli@unimi.it (S.S.); 2 School of Biosciences, University of Nottingham, Sutton Bonington Campus, Loughborough LE12 5RD, UK; ian.hardy@nottingham.ac.uk

**Keywords:** quasi-social parasitoids, xylophagy, mass-rearing system, storage, temperature

## Abstract

**Simple Summary:**

Natural enemies play a fundamental role in the control of invasive insects and may be particularly important in controlling Xylopagous beetle, which are difficult to contain with traditional chemicals as they are usually concealed within wood. Among the natural enemies that have proven effective against these beetles are parasitoid species in the genus *Sclerodermus*. The present article furthers knowledge of *Sclerodermus brevicornis* performance, focusing on the survival capability under different thermal conditions and the longer-term influences of these conditions.

**Abstract:**

Many species of long-horned beetles are invasive pests causing significant economic damage in agro-forestry systems. They spend the majority of their life-cycle concealed inside natural wood or wooden packaging materials and are largely protected from adverse environmental conditions and pesticide sprays. Biological control via parasitoid natural enemies including members of the bethylid genus *Sclerodermus*, has proven effective against some long-horned beetles that are invasive in China. In Europe, the biocontrol potential of native *Sclerodermus* species is being evaluated with a view to developing efficient mass-rearing techniques and then actively deploying them against invasive Asian beetles. Here, we continue evaluations of *S*. *brevicornis* by establishing that groups of females that have already reared offspring to emergence are capable of reproducing subsequent hosts and by evaluating the lifetime reproductive capacity of individual females provided with successive hosts. Additionally, we assess the laboratory shelf-life of adult females stored for different times at different temperatures including cold storage, and then assess the post-storage reproductive performance of groups of females provided with a single host. We found that adult female longevity declines with increasing storage temperature and that most aspects of subsequent performance are negatively affected by high temperatures. The adaptability to low temperature storage enhances the suitability of *S*. *brevicornis* to mass-rearing programs and thus biocontrol deployment.

## 1. Introduction

International trade in ornamental plants and wood packaging materials has increased the unintentional translocation of exotic xylophagous species over long distances [1,2,3]. Xylophagous insects including longhorn beetles (Coleoptera: Cerambycidae) are among the most successful exotic invaders [4]. Many exotic longhorn beetles have been detected in Europe and can spread between countries [5,6]. Despite phytosanitary regulations aiming to reduce this spread [7,8], the rate of new detections is increasing [9] as a consequence of the continuous movement of goods and people and global climate change [10,11,12]. The invasion success of longhorn beetles is largely due to them spending the majority of their life-cycle concealed inside galleries excavated in natural wood or wooden packaging materials [13], and thus being protected from both adverse conditions during transport and many phytosanitary control measures such as pesticide sprays [14,15,16].

Many of the most dangerously invasive cerambycids belong to the tribe Lamiinae. Of particular note are the Citrus longhorn beetle *Anoplohora chinensis* (Forster) and the Asian longhorn beetle *A. glabripennis* (Motshulsky), for which rigorous quarantine measures and regulations have been applied [13,17,18]. Despite the measures adopted against it, *A. chinensis* has been present in Italy since its first detection 20 years ago, albeit with a restricted distribution. Regulations have developed from a policy of eradication to one of containment [19]. Other exotic Lamiinae such as the Yellow spotted longhorn beetle, *Psacothea hilaris hilaris* (Pascoe) were first included in the European and Mediterranean Plant Protection Organization (EPPO) lists, but were later removed because of their limited spread and their association with hosts of minor economic importance within Europe [20], even though infested plants may rapidly be killed [21,22]. Further members of the Lamiinae are sawyer beetles in the genus *Monochamus,* which includes species that are native to Europe and may function as vectors of the pine wood nematode [23], a pest of major economic importance.

Biological control has been widely investigated as the priority method for the containment of exotic pests in general and of pests of woody plants in particular, and many studies have considered the potential of both introduced and autochthonous parasitoids to control invasive pests [24,25,26,27,28,29,30,31]. Most parasitoids attacking longhorn beetles belong to the Hymenoptera [32,33]; some attack hosts at the eggs stage (species in the families Eulophidae [34], Encyrtidae [35,36,37] and Pteromalidae [38]) and some attack hosts during the larval stage (species in the families Braconidae [39,40,41,42], Ichneumonidae [42,43] and Bethylidae [44,45]). Only a minority of this array of parasitoids of exotic longhorn beetles is likely to be effective within biocontrol programs as they must ideally be both suitable for mass production and release and subsequently able to detect and access the target pest inside its galleries [41,46].

Stimulated by successes in agro-forestry systems in China [47], there is growing interest in the use of parasitoids in the bethylid genus *Sclerodermus* Latreille. These are idiobiont ectoparasitoids [48,49], typically attacking coleopteran larvae, mainly wood-boring beetle larvae (Cerambycidae, Scolytidae, Anobiidae and Bostrichidae) [50,51]. Their flattened body and their strong mandibles allow them to enter narrow places in search of hosts, excavating in the frass of pre-existing galleries bored in the wood by the xylophagous beetles [50,52,53,54,55]. Multiple ‘foundress’ females may attack a single host individual, which enhances suppression of large hosts [56]. Females oviposit onto paralyzed hosts communally and exhibit cooperative brood care, without apparent division of labor, tending the offspring until pupation into cocoons and subsequent emergence [48,49,50,56,57,58,59]. Such ‘quasi-sociality’ [60] is an unusual life-history characteristic among parasitoid hymenopterans [61,62]. *Sclerodermus* species also show sexual dimorphism, and strongly female biased sex-ratios: males are smaller and emerge before females, only surviving a few days to mate with the newly maturing females while still in their cocoons [49,63,64]. Males are normally alate, though some male aptery is observed [65]. In contrast, females are larger and survive much longer. Females are typically apterous but some are alate, with the percentage of aptery determined by photoperiod and temperature [59,66]. Wing polymorphisms are thought to encourage the local retention of apterous individuals within infested areas and allow the dispersal of alate forms to sites of further infestation [66,67,68,69,70].

In China, several species of *Sclerodermus* are used in forest pest management programs [47,55,63,71,72,73]. They are released for the control of the pine sawyer beetle, *Monochamus alternatus* Hope (Coleoptera: Cerambycidae) [47,50,74,75], the oak longhorn beetle, *Massicus raddei* (Blessig) (Coleoptera: Cerambycidae) [50,73,76,77], and the emerald ash borer, *Agrilus planipennis* Fairmaire (Coleoptera: Buprestidae) [50,76]. In Europe, the biocontrol potential of *Sclerodermus* species has been relatively little examined. However, following its detection in association with *Anoplophora* spp. [78] and with *Psacothea hilaris hilaris* [79], a program of research into the biology of *Sclerodermus brevicornis* has begun to explore its potential for application in biological control programs as well as to better understand the unusual quasi-social life-histories exhibited within the genus [45,61,62,63]. Here we provide further evaluations of the reproductive performance and mass-rearing potential of *Sclerodermus brevicornis*.

The feasibility of large-scale rearing of *S. brevicornis* on the natural host *P. h. hilaris,* and on the exotic invasive species *A. chinensis* and *A. glabripennis,* was first evaluated by Lupi et al. [45], who showed that it could successfully attack all three species and that rearing was more efficient when large sized host larvae were provided, often generating over 100 offspring per host. However, the trialed beetle hosts are themselves laborious to obtain and rear in the laboratory. Larvae of the rice moth, *Corcyra cephalonica* Stainton (Lepidoptera: Pyralidae), which is considerably more straightforward to laboratory-rear than cerambycid species, were thus trialed as hosts and found to be suitable, although typically less than 10 *S*. *brevicornis* were produced per host [65]. Using either *C. cephalonica* or *P. h. hilaris,* recent experiments have explored the basis of cooperative host attack and reproduction in *S*. *brevicornis*, finding that host attack occurs earlier when groups of females are larger and also when they are relatives [61,62]. However, groups of related females ultimately produced similar [61] or smaller [62] groups of offspring. It was also shown that when an individual *S*. *brevicornis* female attacks a large *P. h. hilaris* larvae, the parasitoid commonly does not survive, often being bitten into pieces [62].

In this study, we continue the evaluation of *S*. *brevicornis* rearing systems, using mainly *P. h. hilaris* as the host. First, we asked whether groups of females that had already reared offspring to emergence were still capable of attacking and reproducing on subsequent hosts. Second, we evaluated the lifetime reproductive capacity of individual females provided with successive hosts. Third, we assessed the laboratory shelf-life of adult females stored for different times at different temperatures including cold storage. Fourth, we assessed the post-storage reproductive performance of groups of females provided with a single host.

## 2. Materials and Methods

### 2.1. Rearing Psacothea hilaris hilaris Host Stock

*Psacothea hilaris hilaris* have been reared in a climatic chamber (26.0 ± 0.5 °C, L16:D8 photoperiod, 70 ± 5% R.H.) at the laboratories of the Department of Food, Environmental and Nutritional Sciences of the University of Milan since 2006, when the insect was first detected in northern Italy [20,79]. To avoid genetic bottlenecks, the laboratory population has been augmented once per year with individuals found in areas were the pest has established [20,80]. In order to maintain the laboratory rearing and provide enough specimens for the experiments, newly emerged adults were sexed based on the observations of the sexual dimorphism in the antennae and last ventrite [80]. The rearing system followed methods given in Favaro et al. [81], with separate protocols for adults and larvae. Pairs of adults (male + female) were transferred into aerated plastic cages (50 × 25 × 40 cm) with a maximum of ten pairs per cage. The adults were able to move freely within these cages, feed on the bark of fig twigs that were provided, mate, and oviposit into the twigs. The fig twigs were replaced three times per week and, once removed from the cages with *P. h. hilaris* adults, kept in aerated plastic boxes (20 × 30 cm × 10 cm height), placed in the same climate controlled chamber as the adults to allow offspring development. After 20 days, infested twigs were debarked and larvae were collected and moved individually into the cups within 24-well plates filled with an artificial diet that allowed the development of *P. h. hilaris* [82]. At least once per week, each larva was provided with fresh diet and transferred progressively, as its size increased, from 12-well plates (plate diameter 2 cm; height 1.5 cm) to 6-well-plates (plate diameter 3.5 cm; height 1.5 cm) and finally into plastic jars (5.5 cm diameter × 3.2 cm height). Lids of well-plates were perforated to allow gas exchange. If larvae were not used for experiments, they were reared in the diet until pupation when they were transferred to a plastic box (95 mm diameter × 40 mm height) until adult emergence and full cuticle sclerotization. Matured adults were sexed and placed in pairs into adult rearing plastic cages, as above.

### 2.2. Rearing Corcyra cephalonica Host Stock

As *P. h. hilaris* cultures are time-consuming to maintain, for assessment of the ability of the parasitoid to reproduce, we followed Abdi et al. [65] and used *C. cephalonica* as factitious hosts. Larvae were reared on an artificial diet used to rear Lepidoptera associated with stored post-harvest products [83]. About two-hundred *C. cephalonica* eggs were added into a Petri dish (15 cm diameter × 2 cm height) half-filled with diet. Larvae that were not used in the experiments were allowed to pupate and eventually emerge as adults. Adults were collected and placed in a glass jar (15 cm diameter × 25 cm height), closed by a mesh held by an elastic band, where they could move, mate, and oviposit. Once per week, eggs were collected and placed onto fresh medium, as above.

### 2.3. Rearing Sclerodermus brevicornis Parasitoid Stock

A laboratory rearing system for *S. brevicornis* was set up in 2011 starting with specimens collected in the north of Italy (45°49′ N, 9°13′ E) where they were found in association with *P. h. hilaris* [84]. Subsequent findings in the field allowed the enrichment of the stock culture [45]. Rearing was carried out in a climate chamber at 23 ± 1 °C, 16L:8D, and RH 60 ± 5%. Once per week, *P. h. hilaris* larvae were used as hosts for *S. brevicornis* development. As host size influences the number of offspring produced, medium and large size hosts were chosen (following Lupi et al., [45]) in order to obtain large numbers of adult wasps and maximize the probability of broods containing adult males as well as females. Larvae were placed individually in plastic jars (5.5 cm diameter × 3.2 cm height) and female wasps (foundresses) added according to the protocol in Lupi et al. [45]. When the host was paralyzed by the foundresses, the lid was micro-perforated with a needle to allow ventilation. Rearing containers were checked periodically until offspring emergence. Emerged females were used either as new foundresses to sustain the stock rearing system or in experiments.

### 2.4. Capacity of Multi-Foundress Groups to Utilize Successive Hosts

To investigate if groups of *S. brevicornis* females could reproduce on several successive hosts, 90 newly emerged females were collected from the laboratory culture and formed into groups of three foundresses. Each group was provided with a single *P. h. hilaris* larva with a mean weight of 0.31 ± 0.18 g (weighed with a TE64 digital precision balance (Sartorius, Goettingen, Germany) in a closed plastic container (5.5 cm diameter × 3.2 cm height). Host larvae were observed twice per week and any dead (dried or decayed) hosts were replaced. When foundresses were observed to have paralyzed the host, the container lids were micro-perforated with a needle to provide ventilation. Once the offspring pupated, any living foundresses were transferred to new containers and, as a group, presented with a fresh host. When one or two foundresses had died before their offspring pupated, new groups of three individuals were formed from among the survivors. The identity of original groups was taken into account by including a random factor in the analysis, but crossover between groups due to female mortality was not (we thus regarded the statistical conclusions as heuristic rather than definitive). This procedure was repeated until no more surviving foundresses were available. Data on the occurrence of oviposition and the time taken to oviposit were collected twice per week.

### 2.5. Reproductive Capacity of Individual Females

To assay the lifetime reproductive capacity of individual females, 120 newly emerged females were placed individually in vials (1.5 cm diameter × 10 cm length) each containing a *P. h. hilaris* larva (mean weight = 0.30 ± 0.17 g) and then observed twice per week. If parasitoid offspring were produced and reached the pupal stage, the foundress was transferred to a new vial containing a fresh host. This was repeated until the foundress died. The age of the foundress at host presentation, the occurrence of offspring emergence, the timing of reproductive events, the number of offspring per larvae, the total number of offspring per foundress, and the sex ratio were recorded.

### 2.6. Longevity of Sclerodermus brevicornis Females Stored at Different Temperatures

The longevity of newly emerged *S. brevicornis* females was evaluated at four constant temperatures: 4.5 °C, 23 °C, 28.5 °C, and 34 °C (all ± 1 °C). We chose 4.5 °C because it is the temperature of laboratory refrigerators used to cold-store insects. We chose 23 °C as it was thought from prior experience to be within the optimal temperature range for rearing *S. brevicornis*. The two remaining treatments, 28.5 °C and 34 °C, were chosen to evaluate parasitoid tolerance to higher temperatures. The wasps were kept in darkness in the refrigerator at 4.5 °C while at other temperatures, the photoperiod was 16L:8D. In all treatments, females had developed on *P. h. hilaris* hosts and were not provided with food as adults. There were 200 replicates for each temperature treatment. To facilitate the monitoring of wasp longevity, replicate females were grouped into batches of 10 and placed inside transparent plastic boxes (5.5 cm diameter × 3.2 cm height) closed with lids. Females held at ≥23 °C were observed three times per week and any *S. brevicornis* that had died were counted and removed. At 4.5 °C, females were checked once per week to minimize temperature fluctuations, given that to evaluate their viability (they remained motionless at 4.5 °C) they had to be brought up to room temperature for 30 min.

### 2.7. Post-Storage Reproductive Performance of Sclerodermus brevicornis Females

The influence of storage temperatures of 4.5 °C, 23 °C, and 28.5 °C on *S. brevicornis* performance was investigated using the same conditions and replication as in the longevity experiment. On the first day and once per week thereafter, 30 females from within each treatment were chosen at random. Ten groups of three foundresses were formed and placed in a vial (10 cm long × 1.2 cm diameter) stoppered with a tampon. This procedure was repeated until half of the original females per treatment had died (DR_50_). This generated, for each temperature treatment, sets of replicates that had been held at that temperature for different periods of time (T_0_ = just emerged, T_1_ = one week, up to a maximum of seven weeks, T_7_). We converted information on each combination of storage temperature and storage time into a single parameter for statistical analyses by using the physiological time-scale of day-degrees (number of days at a temperature, above a threshold temperature, multiplied by that temperature [85]), assuming a threshold temperature of 0 °C.

Within each temperature treatment, vials containing groups of females were then kept under the same conditions, except for vials containing females that had been held at 4.5 °C, which were transferred at 23 °C and presented with a *C. cephalonica* larva (mean weight = 0.0389 g ± 0.04 S.E. (Standard Error), following Abdi et al. [65], weighed with a Sartorius TE64 digital precision balance). Foundress groups were observed three times per week under a stereo stereomicroscope (Wild Heerbrugg M5A) (Leica Geosystems GmbH, Heerbrugg, Switzerland) to observe paralysis of the hosts, oviposition, and hatching of the eggs. We recorded the incidence and timing of host paralysis, oviposition, hatching, pupation, and adult emergence. Once any offspring had pupated, the foundresses were removed from the vials. Emerged offspring were stored in 70% alcohol solution, and later we recorded under the stereomicroscope the sexual composition of the broods and the numbers of winged and wingless males and females.

### 2.8. Statistical Analysis

The influences of experimental conditions on *Sclerodermus brevicornis* life-history parameters were chiefly explored using generalized linear modeling [86,87] in the *GenStat* statistical package (v19.1, VSN International Ltd., Hemel Hempsted, UK). Logistic models, assuming binomial or quasi-binomial errors, were used for the analysis of proportional response variables, log-linear models, assuming quasi-Poisson errors, for the analysis of integer response variables, and models assuming Gamma errors for analysis of developmental times. This approach adopts assumed error distributions (from within the exponential family) that are likely to match the natural patterns in different types of data and then allows further adjustment via empirically estimated scaling parameters, retaining a Type I error rate of approximately 5% without the need for prior transformation [86,87,88,89]. Hypothesis testing was carried out using backwards elimination of explanatory variables (experimental conditions and other measured parameters, treated as fixed effects) from initial models and by aggregation of factor levels to find minimum adequate statistical models via likelihood-ratio tests [86,88]. When individual females or groups of females were presented with several hosts in sequence, the identity of the individual or group was included as a random factor (generalized linear mixed models, GLMM) to avoid pseudo-replication. We give the percentage deviance explained (%Dev) as a descriptor analogous to *r*^2^.

Survival time data were analyzed using parametric cohort survival analyses [86,89]. A Weibull model, with a time-dependent hazard function, provided a better initial description of the data than did an exponential model, so the influence of temperature on longevity was then explored by fitting temperature as a factor into the Weibull model, with each female treated as an independent replicate [86,89].

## 3. Results

### 3.1. Capacity of Multi-Foundress Groups to Utilize Successive Hosts

Groups of females were able to paralyze and oviposit on successive *P. h. hilaris* hosts. The vast majority (93%) of *Sclerodermus brevicornis* groups were able to oviposit on the first host, but only 13% of groups reproduced a fourth time and this was also the maximum number of group-ovipositions observed (Table 1). While the cumulative probability of oviposition declined significantly across successive hosts (logistic GLMM: Wald χ^2^ = 23.33, degrees of freedom (d.f.) = 3, *p* < 0.001), there was no consistent pattern in the probability of oviposition treating each host presentation as a separate event (Table 1).

For groups that oviposited, the time taken to oviposit after being presented with a host was around 20 days for the first host and 25 days for subsequent hosts (Table 1). Timing was significantly influenced by both the host number and the age of the wasps attacking it, with wasps reproducing more slowly as they became older and had previously reproduced on another host (GLMM with gamma errors: Oviposition number: Wald χ^2^ = 20.32, d.f. = 3, *p* < 0.001; Age of wasps at oviposition: χ^2^ = 62.20, d.f. = 1, *p* < 0.001; Interaction: χ^2^ = 41.57, d.f. = 3, *p* < 0.001).

### 3.2. Reproductive Capacity of Individual Females

Individual females were able to reproduce on several successive *P. h. hilaris* hosts. Around one quarter of the females were successful on the first host and only around 1% of females reproduced on three or four hosts (Table 2). Successful females were transferred to fresh hosts when their offspring pupated, and the mean time from host presentation to female transfer declined with host order (Table 2). Around one third of the broods produced consisted of male offspring only, indicating that the mother was unmated. The sex ratios (proportion of offspring that were male) of the remaining broods were strongly female biased (mean = 0.0354, +S.E. = 0.007, −S.E. = 0.006).

**Figure 1:**
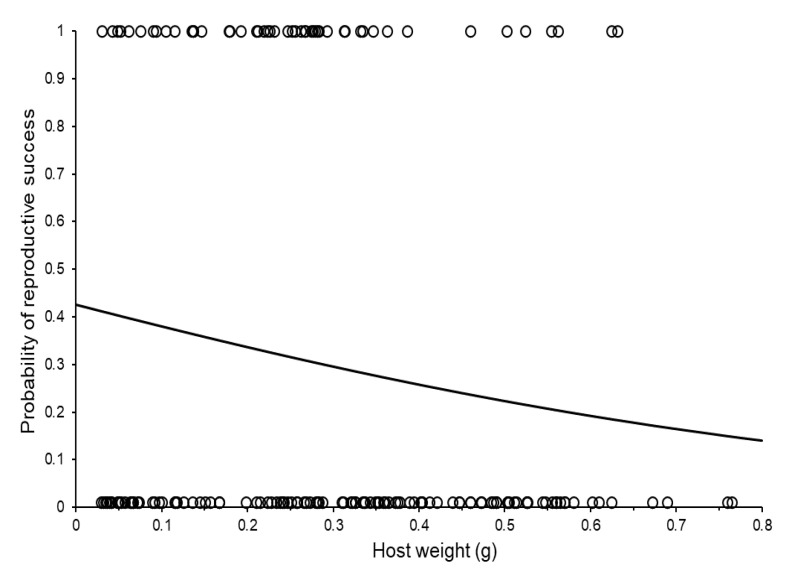
The probability of reproductive success of individual *Sclerodermus brevicornis* females according to the size of the host presented. The fitted curve was estimated using simple logistic regression and (estimated probability = 1/(1 + (1/(antiLog_e_ ((−1.9 × Host weight) − 0.3))). The relationship is marginally non-significant (see main text).

The probability of reproduction on a given host was not significantly affected by host order (logistic GLMM: Wald χ^2^ = 0.48, d.f. = 3, *p* = 0.697), neither by the age of the foundress at host presentation (Wald χ^2^ = 0.81, d.f. = 1, *p* = 0.369), nor by whether the mother was a virgin (as indicated by the sexual composition of previous broods: Wald χ^2^ = 0.00, d.f. = 1, *p* = 0.979). The weight of the host had a marginally non-significant effect on the probability of reproduction (Wald χ^2^ = 3.58, d.f. = 1, *p* = 0.059; note that *p*-value estimates from logistic analyses are not exact, [86]): the trend was that the probability of success declined as host size increased (Figure 1).

**Figure 2:**
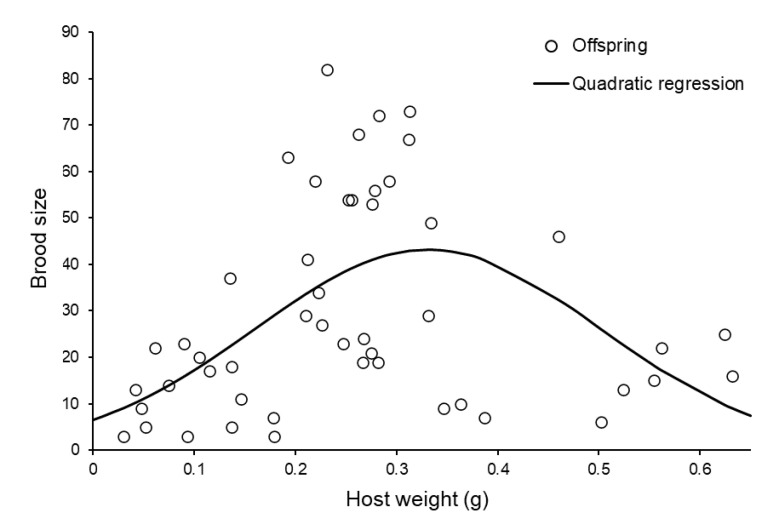
The number of offspring produced by individual *Sclerodermus brevicornis* females according to the size of the host presented. The fitted curve was estimated using log-linear regression including a quadratic term (brood size = antiLog_e_ ((11.37 × Host weight) + (−17.19 × Host weight^2^) + 1.887)).

The number of offspring produced by females that were successful was not affected by the age of the female at the time of host presentation (log-linear GLMM: Wald χ^2^ = 2.48, d.f. = 1, *p* = 0.125), but was affected by the position of the host in the sequence (Wald χ^2^ = 16.55, d.f. = 3, *p* = 0.011), with low production on second hosts and high production on third hosts, and also by whether the mother was a virgin (Wald χ^2^ = 6.50, d.f. = 1, *p* = 0.014), with virgins producing more offspring (virgin mothers: mean = 40.41, +S.E. = 6.45, −S.E. = 5.56; mated mothers: mean = 23.90, +S.E. = 3.59, −S.E. = 3.12). The weight of the host affected offspring production in a significantly curvilinear manner (Host weight: Wald χ^2^ = 1.08, d.f. = 1, *p* = 0.299, quadratic term added after inspection of residuals surrounding the straight-line fit: Wald χ^2^ = 18.07, d.f. = 1, *p* < 0.001, Figure 2).

### 3.3. Longevity of Sclerodermus brevicornis Females Stored at Different Temperatures

**Figure 3:**
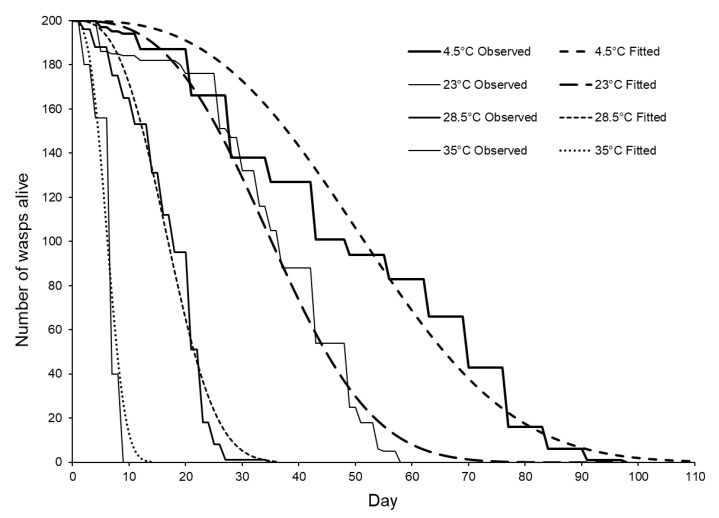
*Sclerodermus brevicornis* adult female longevity at different temperatures. Fitted curves were estimated using parametric cohort survival analysis including age dependence (estimated shape parameter α = 2.87; estimated rate parameters: 4.5 °C = 0.00000842; 23 °C = 0.000025219; 28.5 °C = 0.000208744; and 34 °C = 0.0036849) with the number of females alive on a given day calculated as = 200 × (antiLog_e_ (− (Rate × Day^α^))).

Some *S. brevicornis* lived almost 100 days, but longevity was strongly temperature dependent: survival time decreased with increasing storage temperature (G_4_ = 612.20, *p* < 0.001, %Dev = 70.03, Figure 3, Table 3). Note that the analysis treated each female as an independent replicate even though females were grouped into boxes: as the effects of different temperatures were so clear, we did not consider it likely that pseudo-replication caused Type I error.

### 3.4. Post-Storage Reproductive Performance of Sclerodermus brevicornis Females

**Figure 4:**
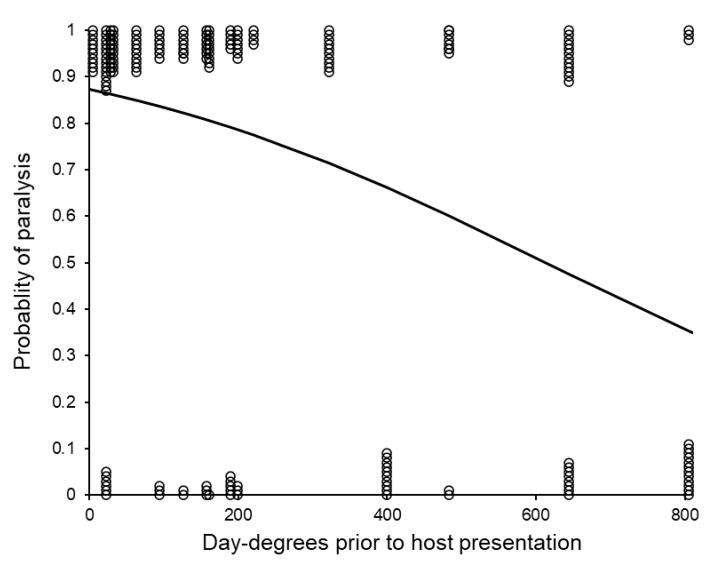
The probability of paralysis by groups of *Sclerodermus brevicornis* females according to the time and temperature (day-degrees) at which they had been stored. Some data points are displaced from their binary values to reduce overlap and illustrate sample sizes. The fitted curve was estimated using logistic regression (estimated probability = 1/(1 + (1/(antiLog_e_ ((−0.003159 × Day-degrees) + 1.935))))).

The host was paralyzed by the *S. brevicornis* females in 73% of replicates. The number of storage day-degrees significantly, and negatively, affected the probability of paralysis (logistic analysis: *G*_1_ = 24.51, *p* < 0.001, %Dev = 13.09, Figure 4). The probability was, however, not significantly affected by the weight of the *C. cephalonica* host (*G*_1_ = 1.05, *p* = 0.304, %Dev = 0.56) or by the temperature at which replicates were maintained post-storage (*G*_1_ = 1.03, *p* = 0.311, %Dev = 0.55) or by any interactions between the three fitted main effects.

Among the replicates in which the host was paralyzed, the proportion of foundresses that had died while others were alive and laying eggs was 0.1691 (+S.E. = 0.0195, −S.E. = 0.0179). The probability of foundress death was not significantly affected by host weight (logistic analysis adopting quasi-binomial errors: *F*_1,131_ = 0.05, *p* = 0.828, %Dev = 0.035), post-storage temperature (*F*_1,130_ = 0.002, *p* = 0.970, %Dev = 0.001), storage day-degrees (*F*_1,132_ =3.75, *p* = 0.055, %Dev = 2.79), or by any interactions between these three main effects (however, the effect of day-degrees was marginally non-significant: the trend was for an increase in mortality with increasing storage day-degrees).

**Figure 5:**
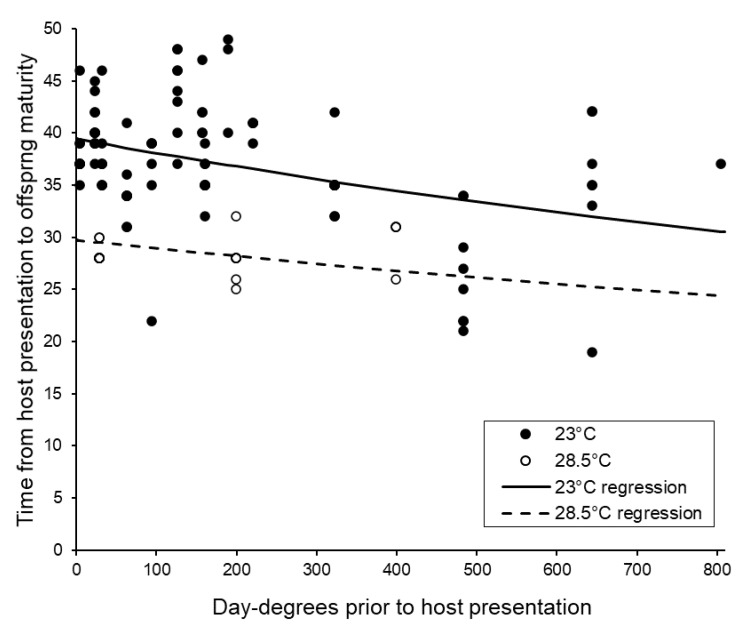
The effects of pre- and post-storage temperatures on the duration of *Sclerodermus brevicornis* reproduction. The fitted curve was estimated using ANCOVA assuming gamma errors and a reciprocal link (for 23 °C, time = 1/((0.00000919 × Day-degrees) + 0.025349); for 28.5 °C, time = 1/((0.00000919 × Day-degrees) + 0.033639)).

The timing of development was affected by both the number of storage day-degrees that were accumulated by the foundresses and by the temperature at which broods were subsequently produced. Most stages of development were faster when the foundresses had accumulated more storage day-degrees, and also when the broods were produced at a higher temperature post-storage (Figure 5, Table 4, see also Table 5).

**Figure 6:**
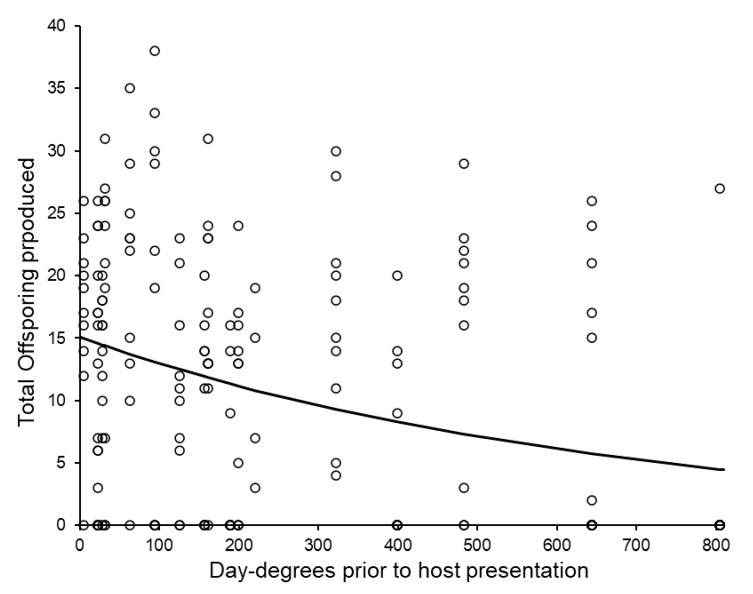
The total number of offspring produced by groups of females according to the number of day-degrees accumulated during storage. The curve was fitted using log-linear regression (Offspring produced = antiLog_e_ ((−0.001504 × Day-degrees) + 2.7146).

The total number of offspring produced by each group of females was not significantly affected by host weight (log-linear analysis adopting quasi-Poisson errors: *F*_1,185_ = 2.80, *p* = 0.096, %Dev = 1.35) or post-storage temperature (*F*_1,186_ = 0.70, *p* = 0.403, %Dev = 0.33), but declined as storage day-degrees increased (*F*_1,137_ = 20.03, *p* < 0.001, %Dev = 9.66, Figure 6, see also Table 6). There were no significant interactions between these three main effects.

**Figure 7:**
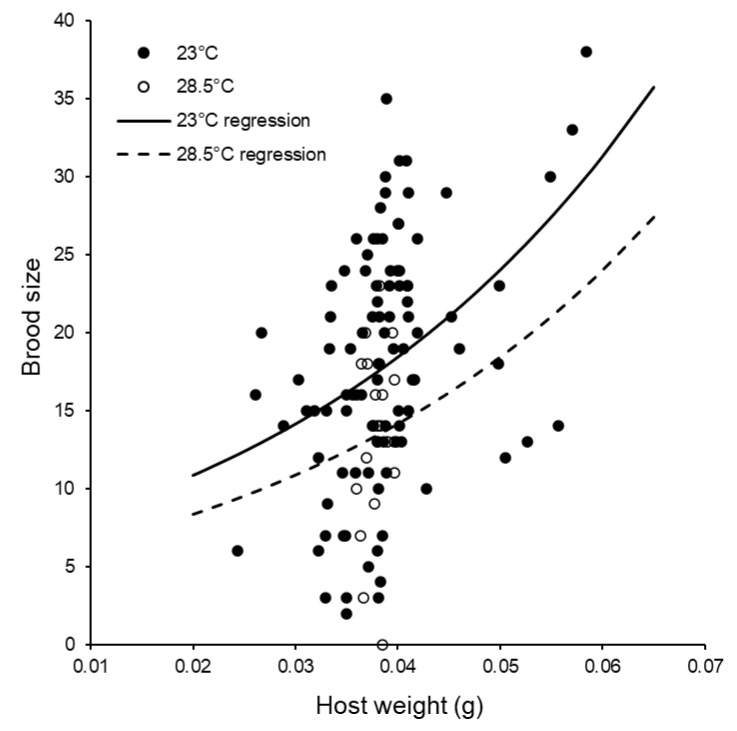
The influences of host weight and post-storage temperature on brood sizes produced by groups of females. The curves were fitted using log-linear ANCOVA (Brood size produced at 23 °C = antiLog_e_ ((26.44 × host weight) + 1.857); Brood size produced at 28.5 °C = antiLog_e_ ((26.44 × host weight) + 1.591)).

Repeating the above analysis with replicates in which foundresses produced no offspring were excluded, we found that brood sizes were not influenced by storage day-degrees (*F*_1,115_ = 0.27, *p* = 0.605, %Dev = 0.19), but were positively affected by host weight (*F*_1,116_ = 14.78, *p* < 0.001, %Dev = 10.64) and were significantly lower at the higher post-storage temperature (*F*_1,116_ = 5.58, *p* = 0.020, %Dev = 4.02, Figure 7, see also Table 6). There were no significant interactions between any of these main effects.

**Figure 8:**
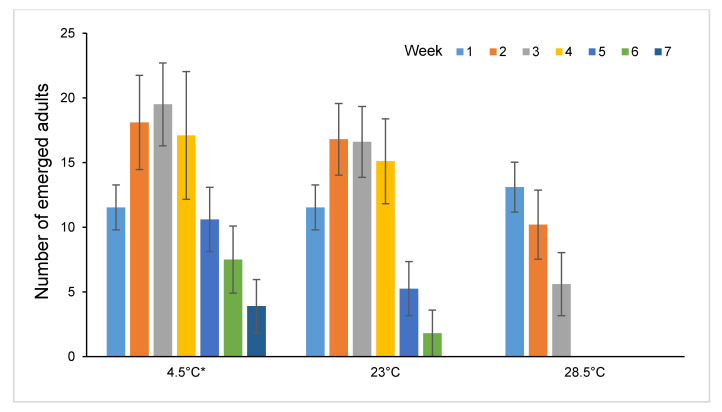
Mean (± standard error, S.E.) adult offspring per host obtained according to foundresses storage time (weeks from emergence) and temperature.

The trends in the mean numbers of emerged adult per larvae in different weeks at each storage temperature condition is shown in Figure 8. The pattern of emergences at 23 °C was similar when foundresses were stored at 4.5 °C or 23 °C: production increased with the age of the ovipositing females, reaching the peak from the second to the fourth week, then decreased progressively with age. At 28.5 °C, production simply declined with foundresses age.

**Figure 9:**
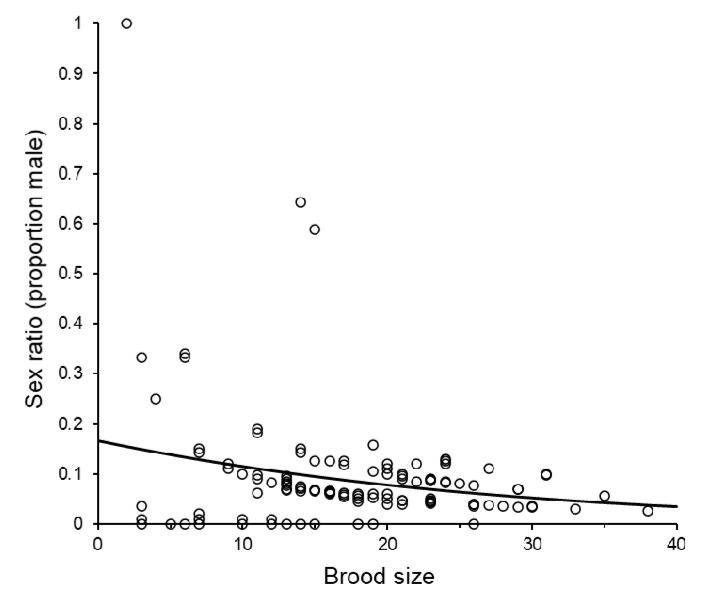
Sex ratios of broods produced by groups of *Sclerodermus brevicornis* females in relation to the size of the brood. Some data points were vertically displaced from their exact proportions to reduce overlap and illustrate sample sizes. The fitted curve was estimated using logistic regression and (estimated probability = 1/(1 + (1/(antiLog_e_ ((−0.0427 × brood size) − 1.616))))).

Brood sex ratios were strongly female biased (proportion of offspring that were male: Mean = 0.0789, +S.E. = 0.0069, −S.E. = 0.0064). Sex ratios were not affected by storage day-degrees (logistic analysis adopting quasi-binomial errors: *F*_1,14_ = 1.98, *p* = 0.163, %Dev = 1.57), the weight of the *C. cephalonica* host (*F*_1,14_ = 1.15, *p* = 0.287, %Dev = 0.91), or by the post-storage temperature (*F*_1,13_ = 0.91, *p* = 0.344, %Dev = 0.72) or by any interactions between the fitted main effects. Sex ratios, however, declined significantly as brood size increased (*F*_1,15_ = 11.30, *p* < 0.001, %Dev = 8.99, Figure 9). The same conclusions were drawn when the analysis was repeated with the single all male brood excluded.

Amongst the adult offspring, there were very few apterous males and very few alate females, yet wing dimorphism in both sexes did occur (Table 7). As there were so few broods containing wingless males or winged females, our statistical conclusions remain tentative. The proportion of female offspring within broods that were winged was typically very low, but also significantly higher when broods were produced at 28.5 °C compared to 23 °C (logistic ANOVA: *F*_1,15_ = 27.33, *p* < 0.001, %Dev = 19.20). For males, the proportion of winged offspring was typically very high, but also significantly higher when broods were produced at 28.5 °C (*F*_1,99_ = 7.98, *p* = 0.006, %Dev = 8.06). Storage conditions of the foundress group also had a significant effect on wing-dimorphism of both males and females (logistic regression, Females: *F*_1,15_ = 4.45, *p* = 0.037, %Dev = 3.87; Males: *F*_1,99_ = 38.84, *p* < 0.001, %Dev = 28.17), so that within a sex, dimorphism occurred only when the foundresses had accumulated few day-degrees in storage.

## 4. Discussion

Successful biocontrol programs are often reliant on mass-rearing and storage of natural enemies for subsequent field release. Mass-rearing systems should ideally be both efficient in parasitoid production and also produce individuals that will perform well [90]. In this study, we evaluated the capacity of adult female *Sclerodermus brevicornis* to be utilized more than once in offspring production, which could clearly increase the per-female reproductive output of mass-rearing units and also possibly overall efficiency. We also evaluated the shelf-life and performance of adult females in relation to temperature.

In terms of the capability of multiple bouts of reproduction (iteroparity), *S. brevicornis* females are clearly able to contribute to several successive broods of offspring, despite post-ovipositional care of each brood taking a long time compared to most parasitoid species. Hu et al. [49] found that in *S. harmandi*, egg laying activity by females was concentrated in the first two days after the commencement of oviposition. This is likely to also be the case in *S. brevicornis* as the emergence of offspring that have developed on the same host are synchronized. The ability to oviposit repeatedly has been previously observed in *S. pupariae*, which has five overlapping generations per years in Tianjin (China) and in which females can oviposit several times [76].

While we observed a maximum of four successively parasitized hosts, the vast majority of parasitoids were unable to successfully attack more than two hosts; we do not recommend any mass-rearing unit to routinely utilize females more than twice, whether held alone or in groups. The low rates of success in the paralyzing hosts, observed when females attempted to reproduce alone, is likely due to the physical dangers females face when attacking hosts, especially hosts that are large. Although the host-size dependency we observed in the current study was marginally non-significant, these risks have been clearly found and quantified in previous studies of *S. brevicornis* and of several congeners [61,62].

In terms of the reproductive output per female, this was highly variable, but with a large upper limit (122 eggs). One candidate explanation for low numbers (<10) of eggs produced is that *S. brevicornis* can perform infanticide on the broods they have been tending when the host is decaying and possibly in other circumstances [45]. We observed a dome-shaped relationship between brood size and host weight: while some studies of *Sclerodermus* have found that brood sizes increase monotonically with host size, others have found similar curvilinear relationships [61,62,91].

Most parasitoids have a relatively short shelf-life [92], but this is not the case in *S. brevicornis*. Casual observations (D. Lupi) have indicated that unfed females can survive up to 40 days at room temperature, but their subsequent reproductive performance has not been evaluated. In our trials, unfed females held at 4.5 °C or at 23 °C were typically able to survive more than a month, with individual longevities of nearly 100 days being reached at 4.5 °C. Females stored at 4.5 °C could be used for offspring production until the sixth week whereas females stored at 23 °C until the fourth week, with a similar number of adults subsequently produced. Thus, storage at 4.5 °C is the best option among the conditions that have currently been evaluated. Cold storage is a technique used to increase insect shelf-life in mass rearing systems [93,94,95] and, provided this does not adversely affect subsequent performance [92], allows individuals to be kept until needed in the laboratory [96,97] or in the field [98,99], thus synchronizing artificial production and release with both the culture of the laboratory hosts and the population dynamics of the target pest [92,100,101]. Despite the advantages of cold storage, there may be some negative effects such as increased mortality caused by physical or metabolic injuries (e.g., osmotic stress or anoxia) or reduced quality due to reduction or elimination of endosymbiont bacterial populations or effects on mobility, responses to chemical cues, learning capacity, fecundity, and offspring sex ratio [102]. *Sclerodermus brevicornis* females appear not to suffer from cold storage *per se*, but post-storage performance tends to decline with storage time at any temperature. In contrast, 34 °C seems to be close to the upper temperature limit for adult survival (and also offspring development; even when paralysis and oviposition occurs at this temperature, laid eggs tend to desiccate and die, D. Lupi, pers. obs.). It is considered that parasitoids and hosts within woody tissues are generally protected from high temperature, and it has also been found that the host *Psacothea hilaris hilaris* survives very poorly at temperatures over 30 °C [82].

Offspring development was more rapid at 28.5 °C than at 23 °C and the timing was also influenced by the storage conditions of the mothers, being typically slower when mothers had been stored at lower temperatures or had accumulated fewer day-degrees before being presented with a host. This suggests that cold-stored or low day-degree females have not matured supplies of eggs sufficiently to be able to oviposit rapidly when presented with hosts. The enhanced speed of reproduction of females kept at 28.5 °C is unlikely to compensate, in mass-rearing terms, for their greatly reduced longevity during storage.

Sex ratios of offspring were strongly female biased and were not influenced by storage conditions or post-storage temperature. Similarly, female based sex ratios have been observed in previous studies of *S. brevicornis* and congeners [45,64,103]. Strongly female biased sex ratios typically enhance the potential of biocontrol agents since it is the females, and not the males, that attack the target hosts [104]. The observed patterns of inter- and intra-sexual wing dimorphism were also very similar to those reported in a prior study of *S. brevicornis* [65]. In congeners, female wing morphology is influenced by photoperiod and temperature [59,66], with more winged females being produced at higher temperatures. We similarly found that higher post-storage temperatures were associated with higher proportions of alate females. Apterous males *Sclerodermus* are rarely reported, but our results also suggest that wing development is promoted by higher temperatures. The accumulation of day-degrees during storage appears to reduce the occurrence of the minority wing-morph in both sexes, although further study will likely be required to confirm this effect. The degree of wing polymorphism is a likely influence on the biocontrol ability of *S. brevicornis* as alate forms will be more able to disperse, while apterous forms will be more confined to the locality of their release.

## 5. Conclusions

In conclusion, although *S. brevicornis* is a quasi-social parasitoid that cares for offspring for considerable periods post-oviposition [61,62,65] (as also observed in congeners [48,58,59]), it is also able to produce further broods once a current brood of offspring has pupated. This ability can be exploited in laboratory mass-rearing programs and will likely also enhance the potential of *S. brevicornis* to suppress invasive pest populations in the field, provided that females naturally leave their maturing broods to forage for further reproductive opportunities. The longevity of stored *S. brevicornis* is also temperature dependent and temperature and storage time have further effects on the reproductive life-history of females post-storage. Overall, this species seems well suited to low temperature storage, an attribute that enhances its suitability for mass-rearing programs and thus biocontrol deployment.

## Figures and Tables

**Table 1:** Probabilities and timing of oviposition on successive hosts by groups of *Sclerodermus brevicornis* females.

Estimated Parameter	Host			
	1st	2nd	3rd	4th
**Cumulative probabilities**				
Mean	0.933	0.767	0.167	0.133
+S.E.	0.034	0.068	0.080	0.075
−S.E.	0.063	0.086	0.058	0.051
**Separate probabilities**				
Mean	0.933	0.821	0.227	0.801
+S.E.	0.034	0.061	0.101	0.124
−S.E.	0.063	0.084	0.077	0.233
**Time to oviposition**				
Mean (days)	19.928	25.52	25.400	25.753
+S.E.	2.549	1.683	2.729	5.236
−S.E.	2.027	1.4815	2.246	3.722

S.E.s are asymmetric around means as they are back-transformed from the logit (proportions) or the reciprocal (timing) scales.

**Table 2:** Probabilities of reproductive success on successive hosts by individual *Sclerodermus brevicornis* females and the timing of transfer between hosts.

Estimated Parameter	Host			
	1st	2nd	3rd	4th
**Cumulative probabilities**				
Mean	0.267	0.092	0.008	0.008
+S.E.	0.042	0.030	0.014	0.014
−S.E.	0.038	0.023	0.005	0.005
**Separate probabilities**				
Mean	0.267	0.297	0.111	1.00 ^†^
+S.E.	0.042	0.080	0.154	
−S.E.	0.038	0.069	0.070	
**Time to transfer to next host**				
Mean (days)	32.134	29.896	26.00 ^†^	-
+S.E.	1.568	2.131		
−S.E.	1.392	2.487		

S.E.s are asymmetric around means as they are back-transformed from the logit (proportions) or the reciprocal (timing) scales. ^†^ Standard errors cannot be calculated as the estimate is based on a single replicate.

**Table 3:** Longevity of starved *S. brevicornis* females stored at different temperatures.

Storage Temperature (±1 °C)	Maximum Longevity (Days)	Mean Longevity (Days ± S.E.)	DR_50_ (Days Until ½ the Females Died)
4.5	98	49.81 ± 1.67 ^a^	43
23	58	36.10 ± 0.95 ^b^	36
28.5	35	17.18 ± 0.46 ^c^	17
34	9	6.54 ± 0.15 ^d^	5

Different letters in the same column indicate significant differences among storage temperature (Dunn’s post-hoc test, *p* < 0.05).

**Table 4:** Effects of pre- and post-storage temperatures on the timing of *Sclerodermus brevicornis* reproduction. Data were analyzed using analysis of covariance (ANCOVA) assuming gamma errors and a reciprocal link-function. N.S. = non-significant effect.

Reproductive Stage	Storage Day-Degrees	Post-Storage Temperature	Interaction
Host presentation to paralysis	Faster after more day-degrees F_1,129_ = 11.05, *p* < 0.001	Faster at higher temperature F_1,129_ = 5.22, *p* = 0.024	N.S. F_1,128_ = 0.24, *p* = 0.625
Host presentation to oviposition	Faster after more day-degrees F_1,125_ = 9.38, *p* = 0.003	Faster at higher temperature F_1,125_ = 20.53, *p* < 0.001	N.S. F_1,128_ = 0.38, *p* = 0.541
Paralysis to oviposition	N.S. F_1,119_ = 0.01, *p* = 0.906	Faster at higher temperature F_1,120_ = 5.96, *p* = 0.016	N.S. F_1,118_ = 0.15, *p* = 0.700
Oviposition to larvae	Marginally N.S. F_1,120_ = 3.74, *p* = 0.056	Faster at higher temperature F_1,121_ = 17.06, *p* < 0.001	N.S. F_1,119_ = 0.07, *p* = 0.797
Larvae to pupation	N.S. F_1,118_ = 1.57, *p* = 0.212	N.S. F_1,117_ = 0.31, *p* = 0.580	N.S. F_1,116_ = 0.38, *p* = 0.536
Pupation to adult emergence	Slower after more day-degrees F_1,112_ = 12.02, *p* < 0.001	Faster at higher temperature F_1,112_ = 80.64, *p* < 0.001	Development slowed more greatly by day-degrees accumulated when temperature higher F_1,112_ = 7.91, *p* = 0.006
Overall host presentation to emergence	Faster after more day-degrees F_1,115_ = 19.03, *p* < 0.001	Faster at higher temperature F_1,115_ = 54.31, *p* < 0.001	N.S. F_1,114_ = 2.58, *p* = 0.111

**Table 5:** Timing of reproduction by groups of *Sclerodermus brevicornis* foundresses following storage at different temperatures (means and standard errors are shown according to storage temperature, irrespective of the time stored at that temperature).

Experimental Conditions		Timing of Reproductive Stages (Days)					
Foundress Storage Temperature (±1 °C)	Offspring Production Temperature (±1 °C)	Host Presentation to Paralysis	Paralysis to Oviposition	Oviposition to Hatching	Hatching to Pupation	Pupation to Emergence	Oviposition to Emergence
4.5	23	4.47 ± 0.39 ^a^	4.24 ± 0.27 ^ab^	6.51 ± 0.4 ^a^	8.04 ± 0.47 ^a^	16.54 ± 0.44 ^a^	30.49 ± 0.56 ^a^
23	23	3.24 ± 0.28 ^ab^	4.77 ± 0.21 ^a^	5.11 ± 0.28 ^b^	6.96 ± 0.44 ^a^	17.11 ± 0.39 ^a^	28.82 ± 0.51 ^a^
28.5	28.5	2.90 ± 0.34 ^b^	3.45 ± 0.30 ^b^	3.71 ± 0.31 ^c^	7.25 ± 0.34 ^a^	11.35 ± 0.52 ^b^	22.30 ± 0.41 ^b^

Different letters in the same column indicate significant differences among developmental times (Dunn’s post-hoc test, *p* < 0.05).

**Table 6:** Adult offspring production according to storage temperature.

Storage Temperature (±1 °C)	Post-Storage Temperature (±1 °C)	Replicates with Reproductive Success (%)	Total Offspring Production (Including Zeros) (Mean ± 1 S.E.)	Brood Size (Excluding Zeros) (Mean ± 1 S.E.)
4.5	23	54%	13.36 ± 1.33 ^a^	19.48 ± 1.11 ^a^
23	23	69%	9.35 ± 1.12 ^b^	17.28 ± 1.15 ^ab^
28.5	28.5	67%	9.63 ± 1.44 ^ab^	14.45 ± 1.03 ^b^

Different letters mean significant differences in number of adults emerged (Tukey’s post-hoc test, *p* < 0.05).

**Table 7:** Wing dimorphism among adult offspring.

Sex	Total Offspring	Alate	Apterous
Male	163	160 (98.16%)	3 (1.84%)
Female	1901	12 (0.63%)	1889 (99.37%)
